# A Frequency-Programmable Miniaturized Radio Frequency Transmitter for Animal Tracking

**DOI:** 10.3390/s21196683

**Published:** 2021-10-08

**Authors:** Jun Lu, Huidong Li, Chuan Tian, Mitchell J. Myjak, Jie Xiao, Brian J. Bellgraph, Samuel S. Cartmell, Zhiqun Daniel Deng

**Affiliations:** 1 Pacific Northwest National Laboratory, Richland, WA 99354, USA; jun.lu@pnnl.gov (J.L.); huidong.li@pnnl.gov (H.L.); chuan.tian@pnnl.gov (C.T.); mitchell.myjak@pnnl.gov (M.J.M.); jie.xiao@pnnl.gov (J.X.); brian.bellgraph@pnnl.gov (B.J.B.); samuel.cartmell@pnnl.gov (S.S.C.); 2 Department of Mechanical Engineering, Virginia Polytechnic Institute and State University, Blacksburg, VA 24061, USA

**Keywords:** animal tracking, radio frequency, circuit

## Abstract

In animal tracking applications, smaller transmitters can reduce the impact of the transmitter on the tagged animal and thus provide more accurate data about animal behavior. By combining a novel circuit design and a newly developed micro-battery, we developed frequency-programmable and more powerful radio frequency transmitters that are about 40% smaller and lighter in weight than the smallest commercial counterpart for animal monitoring at the time of development. The new radio frequency transmitter has a miniaturized form factor for studying small animals. Designs of two coding schemes were developed: one transmits unmodulated signals (weight: 152 mg; dimensions: Ø 2.95 mm × 11.22 mm), and the other transmits modulated signals (weight: 160 mg; dimensions: Ø 2.95 mm × 11.85 mm). To accommodate different transmitter life requirements, each design can be configured to transmit in high or low signal strength. Prototypes of these transmitters were evaluated in the laboratory and exhibited comparable or longer service life and higher signal strength compared to their smallest commercial counterparts.

## 1. Introduction

Radio frequency (RF) transmitters have revolutionized biologists’ understanding of terrestrial and aquatic animal movements [1] since they were first attached to animals about 60 years ago [2]. Radio telemetry is a traditional tool to examine fish behavior [3]. Accurate information on fish movement, for example, is needed to understand the impacts of hydroelectric dams on fish migration and survival so that mitigation techniques can be applied to recover endangered populations or to prevent endangerment in the first place. Radio telemetry has been successfully used to track bats [4], is also being employed to examine seabird interactions at offshore wind farms in Europe [5], and to implement California condor wind power mitigation [6]. However, the use of radio telemetry technology has been limited by the relatively large transmitter sizes that could negatively affect the behavior of the animals to which they are attached [7]. Biologists have been continually seeking smaller transmitters to obtain as-accurate-as-possible data to support decision-making to study small animals and minimize the bias resulted from the tag burden to study small animals [8]. For example, for fish tagging, it is generally accepted that the tag should weigh no more than 2% of the fish’s body weight in air [9]. The American Society of Mammalogists recommended transmitter weight should be less than 5% of the bats’ body weight [10]. Many endangered birds and bats weigh only about 4 g, and thus, a transmitter is required to weigh less than 0.2 g to meet the tag burden requirement [8]. However, the smallest commercially available RF transmitter to date weighs approximately 0.24 g [11].

Despite this need, technological advancements to reduce RF transmitters’ sizes and prolong their service life have been lagging in recent years, largely due to the size limitations of high-frequency generator circuits and the battery. The objective of this study was to design a miniature transmitter that is small enough to be injected into fish and other animals and exceeds the specifications (i.e., smaller size, longer battery life, stronger output, and lower cost) of the smallest commercially available RF transmitter. By designing a novel circuit and taking advantage of a new micro-battery technology [12] recently developed at PNNL, two new frequency programable RF transmitters that are significantly smaller and lighter than the smallest commercial counterparts were successfully developed [13]. In this paper, we report the designs and fabrication process of the new transmitters and the experimental results of the prototypes. The prototypes’ transmitting performance and tag life were compared against two of the smallest commercial RF transmitters. The developed technology would enhance the flexibility of studying fish affected by hydropower operations and bats affected by wind turbine collision mortality by reducing behavioral tag effects on these animals and lowering the animal size thresholds for tagging.

## 2. Materials and Methods

The new RF transmitters consist of three main components: (1) an electronic circuit board containing the controlling circuitry, (2) a custom cylindrical micro-battery that powers the transmitter, and (3) an antenna for transmitting RF signals.

### 2.1. Circuit Board Design

**Figure 1:**
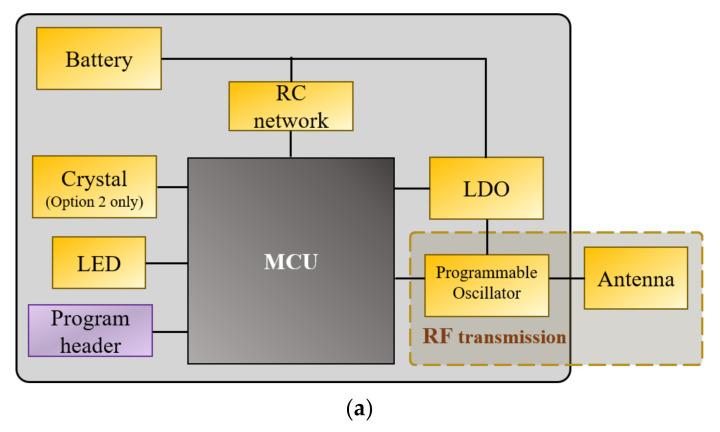

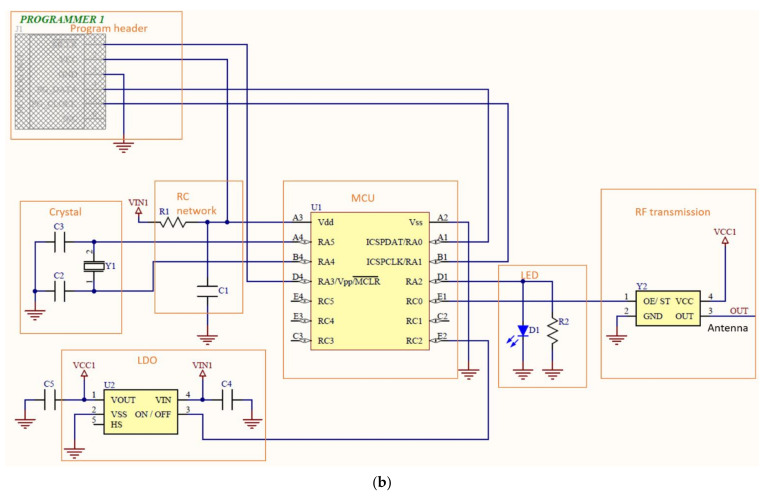
Schematic illustrations of the RF transmitter circuit design: (**a**) circuit diagram; (**b**) circuit schematic.

Figure 1 illustrates the overall electronics design and schematic of the new RF transmitter. Two design variants (option 1 and option 2 hereinafter) of this transmitter were developed based on two different coding schemes: Option 1 transmits unmodulated signals, and option 2 transmits modulated signals, both at a configurable ping rate.

The circuit designs of the two options are nearly identical. Both comprise a miniature microcontroller (U1), a resistor-capacitor (RC, R1, and C1) network, a light-emitting diode (LED, D11), and a low-dropout (LDO, U2) voltage regulator. The only difference is the use of a crystal (Y1) in option 2 to control the modulated RF signals. The microcontroller executes embedded firmware to control various circuit functions, such as turning the programmable oscillator on and off to control the transmitted signal. The components were carefully chosen to achieve the best tradeoffs between performance, power consumption, and physical size and weight.

The new transmitters use one of the smallest microcontrollers, a chip-scale package 8-bit PIC microcontroller PIC16LF1823 [14] with a tiny footprint (2.4 mm × 1.6 mm). This microcontroller provides several features helpful for reducing the overall component count and power consumption. For instance, it has a very low current draw (20 nA in deep sleep mode, or 300 nA in sleep mode with the watchdog timer active). This feature reduces the energy consumption during clock transitions, and the battery voltage drops during signal transmission. The RC network isolates the microcontroller from the battery’s voltage drop and keeps it from experiencing brownout.

The LED provides an optical link for programming through a personal computer. LEDs are conventionally used to convert electrical energy into light as light emitters. It is easy to forget that they are fundamentally photodiodes, and as such, they are light detectors [15]. In these new RF transmitters, rather than generating light when a voltage is applied across its terminals, the LED is used as a photodiode in the converse manner. It generates a voltage across its terminals when exposed to strong light. Using LED as a communication method and wake-up circuit was reported in References [15,16]. However, this is the first time an LED is integrated into a transmitter design as an activation method. Transmitters generally use a Hall-effect sensor, reed switch [17], or phototransistor [18] as the activation and/or programming methods. The footprint of the Hall-effect sensor used in [17] was 2 mm × 2 mm, and the phototransistor used in Reference [18] was 1.6 mm × 0.8 mm. The LED in our transmitter is only 1.00 mm × 0.50 mm, which is less than half of the phototransistor area, so the transmitter size is minimized. When configuring the new transmitters, our programming device utilizes a USB-to-TTL converter circuit and an ultraviolet LED to convert serial commands from a personal computer to a modulated series of “on” and “off” pulses of light, which is then converted back into electrical signals by the LED on the transmitter. These electrical signals are then communicated to the microcontroller through one of the pins of the microcontroller. The above mechanism effectively activates the microcontroller and configures operating parameters such as the RF ping rate and start or end of RF transmission while conserving the fairly limited real estate on the circuit board of such small devices.

Traditional RF signal generating circuits consist of a quartz crystal, an oscillator circuit, and one or more amplifiers [19]. Such configurations require relatively high power consumption and a larger circuit board footprint to implement. Commercial RF transmitters for animal tracking can operate only on specific frequencies, e.g., 48–49, 144–151, and 164–167 [20], 147–168 MHz [11]. To minimize the transmitter size and provide a significantly wider range of frequencies, we developed a novel method to generate RF signals by using a programmable oscillator. This design generates the RF signals by turning on the oscillator and attaching the antenna to the oscillator’s output. Programmable oscillators are generally used as a timing reference for microcontrollers and FPGA systems [21]. To our best knowledge, this is the first RF transmitter to include merely a programmable oscillator for generating the oscillation frequency for RF transmission. The use of a programmable oscillator in the RF transmitter provides the user the flexibility to obtain a desired frequency with short lead time and low cost. It minimizes the circuitry area required for RF signal generation, thus significantly reducing the transmitter size. The programmable oscillator SG-8003CG [22] used in the prototype transmitters has a footprint of only 2.5 mm × 2 mm, much smaller than the traditional RF signal generating circuits. The frequency tolerance of the SG-8003CG can also be programmed to merely 50 ppm at −40 °C to +80 °C, which is comparable to the crystal quartz used in traditional RF signal generating circuits. The input voltage of the SG-8003CG has 3 programmable options, 1.8 V, 2.5 V, and 3.3 V. We program the input voltage to be 1.8 V to lower the current consumption and maximize transmitter service life. 

An LDO regulator is used to output a fixed voltage at 1.8 V to the SG-8003CG to maintain a stable signal strength, which does not change when the battery voltage drops during signal transmission. The programmable oscillator generates a symmetric square wave signal. The Fourier series of a square wave is

(1) $$square\left(t\right)=\frac{4}{\pi }\overset{\infty }{\underset{n=1}{\sum }}\frac{sin\left(nt\right)}{n}=\frac{4}{\pi }\left(\frac{sin\left(t\right)}{1}+\frac{sin\left(3t\right)}{3}+\frac{sin\left(5t\right)}{5}+\cdots \cdots \right)\;\;\;$$

The square wave only contains odd harmonics, and the amplitude is in inverse proportion to the harmonic order *n*. To be compatible with receivers from the major device manufacturers [11,23], the frequency range of the new transmitters was chosen to be 164–168 MHz. The programmable oscillator can be set to 54.7–56.0 MHz for high-strength signals to generate a sine wave at 164–168 MHz using the 3rd harmonic, which consumes a current of 2.7 mA. It can also be set to 32.8–33.6 MHz for low-strength signals using the 5th harmonic, which consumes a current of 2.1 mA.

Compared to option 1, the microcontroller of option 2 uses an external 32.768 kHz clock signal from the added crystal to accurately control the timing of the programmable oscillator so the modulated signals can be precisely generated. The microcontroller also uses this added crystal to calibrate its internal clock. To allow multiple transmitters to broadcast on the same frequency, a pattern of pulses unique to each individual transmitter is used in the option 2 design. For example, transmitters with the same frequency can be differentiated by varying the time period between two lead pulses and varying the time spacing between two trailing pulses [24]. The details of the transmitter coding scheme in this paper are proprietary information provided by Lotek Wireless Inc. [11] through a non-disclosure agreement. 

**Figure 2:**
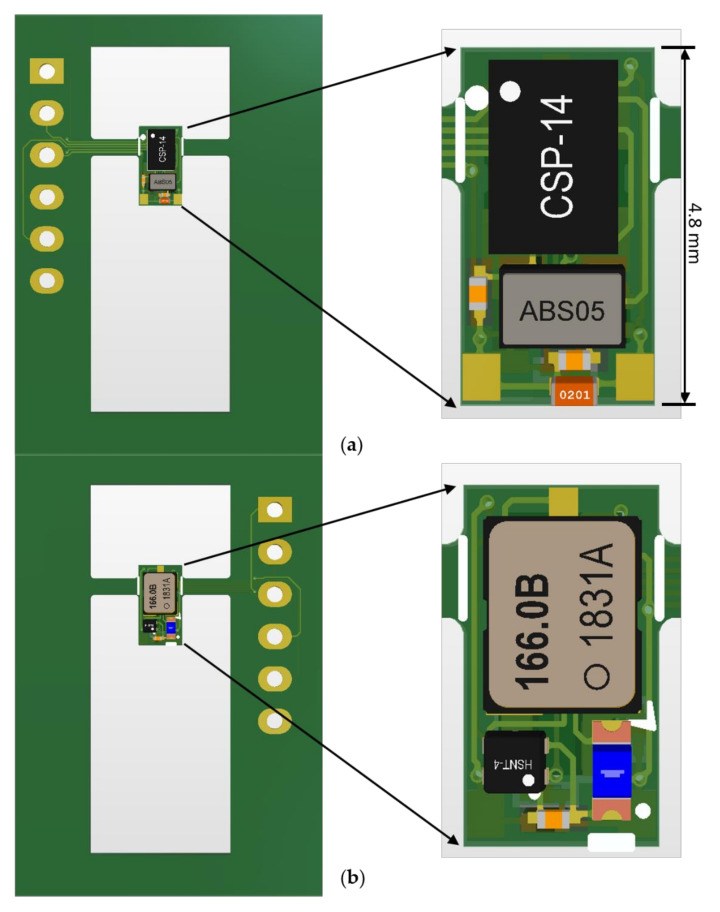
Circuit layout for the option 2 transmitter: (**a**) front side; (**b**) back side.

Figure 2 illustrates the circuit layout for option 2 (right). Both option 1 and 2 designs use a two-layer circuit board of minimal thickness. As shown in Figure 2a, the circuit board is attached to an outer frame at two points. The perimeter of the frame contains two sets of connectors: a 6-pin ICSP header for programming the microcontroller. The circuit board outline is laser-cut inside the frame. To minimize the length of the transmitter, the components on the circuit board are placed as close to one another as possible. The length of the option 2 layout is only 4.8 mm.

### 2.2. Circuit Firmware Design

The microcontroller is programmed in PIC assembly language due to the number of time-critical operations required. For option 1, the firmware turns on the programmable oscillator for RF transmission continuously for approximately 16 ms. For option 2, the firmware turns on and off the programmable oscillator for RF transmission based on Lotek’s coding scheme to generate different transmission codes. The total transmission time is approximately 22 ms.

The microcontroller firmware has several operating modes to dictate the overall operation of the system. The firmware clears all working variables in the “initialization” mode and initializes all parameters to a safe state. In the “waiting” mode, the microcontroller remains in a low-power sleep state and periodically wakes up to check the LED for incoming configuration light signals. In the “hibernation” mode, the microcontroller remains in a low-power sleep state for a set duration prior to transitioning to the “running” mode. In the “running” mode, the microcontroller enables the programable oscillator to start RF transmissions at preset intervals. In the “calibration” mode, the microcontroller calibrates the frequency of its internal timers against the external crystal. Finally, in the “configuration” mode, the microcontroller reads commands from the LED. It sets the operating mode and other parameters such as tag code, transmission period, number of transmissions between calibrations, and hibernation duration. A “debug” mode contains debugging code for development purposes and is not included in the final design.

### 2.3. Battery

Batteries used in RF transmitters have major impacts on the transmitter design and performance, such as transmitter shape and size, service life, transmitting range, etc. Because of the small size and weight of the new RF transmitter, no existing commercial battery on the market could fit into its small package and provide sufficient energy to support the service life needed. The RF transmitter uses a proprietary lithium/carbon fluoride (Li/CFx) micro-battery technology recently developed by PNNL [12] to minimize the device size. The newly designed micro-batteries are intrinsically lightweight and have low impedance, leading to significantly improved electrochemical performance compared with commercial small button cell batteries [12]. The micro-battery used by the transmitter has a capacity of 6 mAh. It has an outer diameter of 2.7 mm and a length of 6 mm and weighs merely 65 mg. Compared to the traditional silver-oxide button-cell batteries commonly used in small commercial radio transmitters, the Li/CFx micro-battery has the advantages of high energy density, high average operating voltage, less variable voltage discharge characteristic, long shelf life, and a wide operating temperature range [12].

### 2.4. Antenna

The antenna is the interface between an RF transmitter and the propagation media. Most fish transmitters are equipped with a monopole antenna (also known as a whip antenna), consisting of a straight length of wire [19]. The optimal antenna of transmitters used in small aquatic animals is often a compromise between efficient radio wave propagation and effects on animal behavior [25]. An experimental comparison of the relative signal strengths of the transmitters over a range of antenna lengths and materials can be used to reduce weight and maximize output power and detection. The antenna used in the prototype RF transmitters was a Teflon PFA (perfluoroalkoxy)-coated stainless-steel wire with a diameter of 38.1 µm. The length of the antenna was 18 cm to achieve the necessary resonance frequency of the transmitter. For a direct comparison, we chose the same antenna length as that of the NTQ-1/2 transmitters by Lotek Wireless Inc., which was approximately one wavelength underwater at the test frequency (164–168 MHz) and had the peak relative signal strengths over a range of antenna lengths, according to [25].

### 2.5. Packaging and Manufacturing

**Figure 3:**
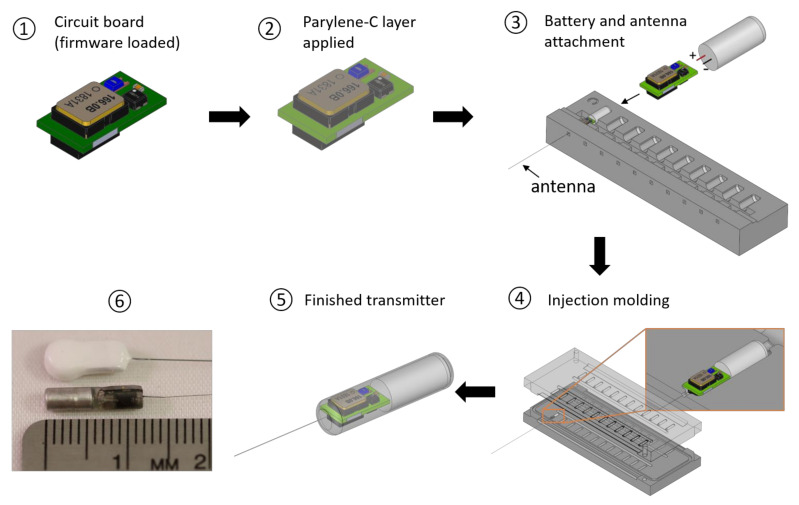
The manufacturing process for the RF transmitter and close-up view: (**1**) manufacture of the thin two-layer PCB with components; (**2**) thin-film parylene-C layer deposition; (**3**) attachment of the micro-battery and antenna; (**4**) encapsulating the transmitter assembly using epoxy; (**5**) the CAD drawing of the Option 2 capsule design; (**6**) Lotek NTQ-2 (top) and the prototype of the modulated RF transmitter (bottom).

For animal tracking applications, the transmitter components (antenna, battery and circuit board) must be assembled and encapsulated into a package for use in animals. The manufacturing process of the RF transmitter involves a combination of printed circuit board (PCB) manufacturing steps, component placement and solder bonding, thin-film deposition, and encapsulation procedures (Figure 3). The transmitter uses a series of small-footprint, high-performance, low-power, off-the-shelf electronic components. Our choices of commercially available electronics and manufacturing protocols ensure the functional reproducibility of the devices and alignment with low-cost volume manufacturing and permit further extended functionalities of the platform with other sensors and peripherals appropriate for relevant monitoring applications. To fabricate the prototype transmitters, a 25-µm thick Parylene-C coating was finally applied to the circuit board to make it waterproof and corrosion-resistant. The antenna was then soldered to the circuit board. The micro-battery was attached to the circuit board by soldering with the help of a 3D-printed jig which aligns the battery with the circuit board. The transmitter assembly was then encapsulated with EPO-TEK 301 epoxy (Epoxy Technology Inc., Billerica, MA, USA) through an injection molding process. After being removed from the mold, the transmitter was mildly polished using a Dremel rotary polishing tool to eliminate any sharp edges or burrs on the transmitter body. Dozens of the transmitters can be produced at a time with a single injection mold. Figure 3e shows the CAD drawing of the option 2 capsule design, and Figure 3f shows a prototype of the option 2 transmitter side by side with a Lotek NTQ-2 transmitter [11]—one of the smallest commercial RF transmitters in 2016 for fish tracking. As one of the design goals of the new RF transmitter was the ability to inject them into fish, the prototype transmitters had a cylindrical body with a diameter of 2.95 mm to be compatible with a 9-gauge needle. The option 1 transmitter was 11.22 mm long and weighed 152 mg. Because of the addition of the crystal, option 2 was 11.85 mm long and 160 mg in weight, slightly longer and heavier than option 1.

## 3. Results

### 3.1. Transmitter Signal Strength Testing

Signal strengths of the prototypes of the two design options were compared against that of the Lotek NTQ-2. The test was performed outdoors to minimize the effects of other electromagnetic background noises. The test transmitters were placed about 10 cm apart and paralleled to one another. The signal receiver (Sigma Eight Orion receiver [26] with an omnidirectional whip antenna) was located about 6 m away. The receiver antenna was arranged perpendicular to the transmitter antennas. The transmitters and the receiver antenna remained at their locations during the test. Both high- and low-signal-strength variants of the option 2 prototype were tested to compare with the Lotek NTQ-2, which uses the same transmission scheme. Both prototype transmitters’ signal strengths were consistently about 10 dB stronger (−76 and −77 dBm for the high and low-signal-strength configurations, respectively) than the Lotek NTQ-2′s (−88 dBm). The higher signal strength may have partially resulted from the higher driving voltage to the antenna in our prototype transmitters than in NTQ-2. Our transmitter circuit used the programmable oscillator to generate the driving voltage, which swings in full CMOS voltage output range across the antenna. The peak-to-peak value of this voltage is higher than what the ASIC in the NTQ-2 can generate from its 1.55 V silver oxide battery.

### 3.2. Transmitter Life Testing

During RF signal transmission, the average voltage for both designs measured at the battery terminals was about 2.5 V. For option 1, the transmission pulse duration was 16 ms for each transmission. At the low-signal-strength setting, the average current draw was measured to be 2.1 mA, and the energy consumption was calculated to be 84 µJ per transmission. At the high-signal-strength setting, the average current draw was 2.7 mA, which led to energy consumption of 105 µJ per transmission. For option 2, the total pulse duration was about 22 ms. At the low-signal-strength setting, the average current draw was 2.1 mA, and the energy consumption was 116 µJ per transmission. At the high-signal-strength setting, the current draw was 2.7 mA, leading to energy consumption of 149 µJ per transmission. 

The service life of the transmitters can be estimated using the total energy of the micro-battery and considering the energy loss due to the static leakage current of the circuit. Because the energy of the micro-battery is consumed by signal transmission throughout the service life of the transmitter and by the quiescent current that constantly flows through the circuit, the service life T can be estimated as follows:

(2) $$T=\frac{V*\frac{\frac{C}{1000}}{24}}{\frac{E_{trans}}{t}+V*I_{s}}\;$$

where:

V—the battery voltage in volts,
C—the battery capacity in mAh,
$E_{trans}$—the energy consumed by each transmission,
$I_{s}$—static current,
t—the signal period (ping rate),
T—the service life in days.

It is worth noting that the projected service life based on Equation (2) is a conservative estimate because the battery’s voltage gradually decreases as it discharges, which consequently causes a gradual reduction of $E_{trans}$ over time.

The actual service life of the option 1 prototype transmitters was tested at a 3-s ping rate (Table 1). The test results were consistent with or higher than the projected value obtained using Equation (2).

Table 2 compares the sizes and weights of the variants of the new transmitters and those of the Lotek NTQ-1 and 2. The service life estimates for the new transmitters presented in this table were based on a measured static current value of 0.5 µA. Compared to the Lotek transmitters, the new cylindrical RF transmitters provide significant reductions (~40%) in weight and width and have just a slightly longer (~10–20%) body.

Combining these properties permits biologists and ecologists to study smaller species for a longer duration of time, which has not been possible with existing devices.

## 4. Conclusions

Two miniature RF transmitters for tracking small aquatic, airborne, or terrestrial animals were designed, prototyped, and evaluated. For the first time, a programmable oscillator is used for generating signal frequency, and a LED is used as the activation method. The transmitters are powered by a newly developed cylindrical micro-battery with a significantly higher energy density than that of traditional silver-oxide button-cell batteries. The pilot laboratory evaluation demonstrated that the prototype RF transmitters have stronger signal strength, are 40% smaller and lighter in weight and exhibit comparable or improved service life than the smallest commercial RF transmitter for animal tracking.

## 5. Patents

A patent resulting from the work reported in this manuscript was issued in March 2019 patent (U.S. No. 10,236,920).

## Figures and Tables

**Table 1:** The Prototype RF Transmitter Life Testing Results.

PNNL Option 1 (3 s Ping Rate) Transmitter	Calculated Life (Days)	Measured Life (Days)
Low-signal-strength Transmitter (test sample 1)	23	30
Low-signal-strength Transmitter (test sample 2)	23	24

**Table 2:** Comparison of Lotek and PNNL Prototype Transmitters.

Transmitter	Size (mm) (w*h*l)	Weight (Air, mg)	Calculated Life (PRI, Days)			
			2 s	5 s	10 s	60 s
Lotek NTQ-1	5*3*10	260	10	21	33	59
Lotek NTQ-2	5*3*10	310	16	33	52	94
PNNL Option 1 Low signal strength	Ø 2.95*11.22	152	15	37	60	245
PNNL Option 1 High signal strength	Ø 2.95*11.22	152	12	24	56	217
PNNL Option 2 Low signal strength	Ø 2.95*11.85	160	11	22	52	205
PNNL Option 2 High signal strength	Ø 2.95*11.85	160	8	21	41	176

## Data Availability

The data presented in this study are available on request from the corresponding author. The data are not publicly available due to restrictions.
